# Continuous Increase of Cardiovascular Diseases, Diabetes, and Non-HIV Related Cancers as Causes of Death in HIV-Infected Individuals in Brazil: An Analysis of Nationwide Data

**DOI:** 10.1371/journal.pone.0094636

**Published:** 2014-04-11

**Authors:** Adelzon A. Paula, Mauro Schechter, Suely H. Tuboi, José Claudio Faulhaber, Paula M. Luz, Valdiléa G. Veloso, Ronaldo I. Moreira, Beatriz Grinsztejn, Lee H. Harrison, Antonio G. Pacheco

**Affiliations:** 1 Programa de Computação Científica, Fundação Oswaldo Cruz, Rio de Janeiro, Brasil; 2 Projeto Praça Onze, Hospital Escola São Francisco de Assis, Universidade Federal do Rio de Janeiro, Rio de Janeiro, Brasil; 3 GlaxoSmithKline, Rio de Janeiro, Brasil; 4 Instituto de Matemática e Estatística, Universidade do Estado do Rio de Janeiro, Rio de Janeiro, Brasil; 5 Instituto de Pesquisa Clínica Evandro Chagas, Fundação Oswaldo Cruz, Rio de Janeiro, Brasil; 6 Infectious Diseases Epidemiology Research Unit, Graduate School of Public Health and School of Medicine, University of Pittsburgh, Pittsburgh, Pennsylvania, United States of America; Infectious Disease Service, United States of America

## Abstract

**Introduction:**

After antiretroviral therapy (ART) became available, there was a decline in the number of deaths in persons infected with HIV. Thereafter, there was a decrease in the proportion of deaths attributed to opportunistic infections and an increase in the proportion of deaths attributed to chronic comorbidities. Herein we extend previous observations from a nationwide survey on temporal trends in causes of death in HIV-infected patients in Brazil.

**Methods:**

We describe temporal trends in causes of death among adults who had HIV/AIDS listed in the death certificate to those who did not. All death certificates issued in Brazil from 1999 to 2011 and listed in the national mortality database were included. Generalized linear mixed-effects logistic models were used to study temporal trends in proportions.

**Results:**

In the HIV-infected population, there was an annual adjusted average increase of 6.0%, 12.0%, 4.0% and 4.1% for cancer, external causes, cardiovascular diseases (CVD) and diabetes mellitus (DM), respectively, compared to 3.0%, 4.0%, 1.0% and 3.9%, in the non-HIV group. For tuberculosis (TB), there was an adjusted average increase of 0.3%/year and a decrease of 3.0%/year in the HIV and the non-HIV groups, respectively. Compared to 1999, the odds ratio (OR) for cancer, external causes, CVD, DM, or TB in the HIV group were, respectively, 2.31, 4.17, 1.76, 2.27 and 1.02, while for the non-HIV group, the corresponding OR were 1.31, 1.63, 1.14, 1.62 and 0.67. Interactions between year as a continuous or categorical variable and HIV were significant (p<0.001) for all conditions, except for DM when year was considered as a continuous variable (p = 0.76).

**Conclusions:**

Non HIV-related co-morbidities continue to increase more rapidly as causes of death among HIV-infected individuals than in those without HIV infection, highlighting the need for targeting prevention measures and surveillance for chronic diseases among those patients.

## Introduction

The widespread availability of ART led to an initial abrupt decrease in mortality in individuals infected with HIV, which leveled off in most countries after a few years [1]–[3]. Although causes of death traditionally associated with HIV/AIDS continue to play a prominent role, other conditions, including cardiovascular diseases (CVD), diabetes mellitus (DM), and cancer emerged as frequent causes of death [4]–[8]. In 1996, Brazil became the first developing country to provide free and universal access to ART. We have previously described an increase in causes of death due to conditions generally not associated with HIV infection in Brazil at the local, regional, and national levels [1], [4], [9]. The aim of the present study was to expand our previous analyses of national temporal trends in selected non-AIDS-related causes of death and TB in persons with HIV infection.

## Methods

In Brazil, the death certificate (DC) is a standardized form that is entered into a national electronic database, which is available online without personal identifiers (http://tabnet.datasus.gov.br/tabdata/sim/dados/cid10_indice.htm). Primary, secondary, and contributing causes of death according to the International Classification of Diseases 10th revision (ICD-10) codes are available for all deaths that occurred since 1999.

We compared temporal trends in causes of death for individuals who had HIV/AIDS listed in any field of the DC (ICD-10 codes B20-B24, Z21-[HIV group]) and for those who did not have HIV/AIDS mentioned (non-HIV group). Logistic regression models were fitted with generalized linear mixed-effects models (GLMM) equations. Statistical methods are described elsewhere [9]. Briefly, year of death was treated either as a continuous or categorical variable in the models. In the former case, linear trends are reported as the average variation per year, while in the latter odds ratios (ORs) are used to compare annual changes relative to the baseline year of 1999. Statistical significance was assessed through differences in slopes in temporal trends testing the interaction term between HIV status and year. We conducted five separate analyses in which the outcomes were defined by the presence or absence in any field of the DC of non-HIV-related cancers, external causes, CVD, DM and tuberculosis (Table 1). All analyses were performed in R for Windows 3.0.2 (http://www.r-project.org), using the package ‘lme4’ for GLMM estimation.

**Table:** **Table1.** Definition of groups of disease and ICD-10 codes used in this study.

Disease/group	ICD-10 code	Comments
HIV group	B20-B24, Z21	Not all codes that mention HIV make sense in this context, so Z11.4 was not included among other codes.
Non-AIDS-related cancers	C00-C97 (except C46 and C80-C89)	Excludes Kaposi's sarcoma, immunoblastic lymphoma, Burkitt's lymphoma, and primary brain lymphoma.
External causes	S00-Y98	Includes violent causes, accidents, non-fatal trauma, poisoning and drug abuse.
Cardiovascular diseases	I00-I99 except I46	Excludes cardiac arrest (not a real cause of death).
Diabetes mellitus	E10-E14	
Tuberculosis	A15-A19	

## Results

A total of 12,366,853 deaths were reported among adults 18 years of age or older between 1999 and 2011. Of these, 151,706 (1.23%) had HIV/AIDS reported in any field of the DC. Mean age at death (annual increment) for the HIV and non-HIV groups were 41.6 (0.39) years and 66.9 (0.16) years, respectively; the annual increment during the study period was significantly higher for the HIV group (p<0.001).

**Figure 1:**
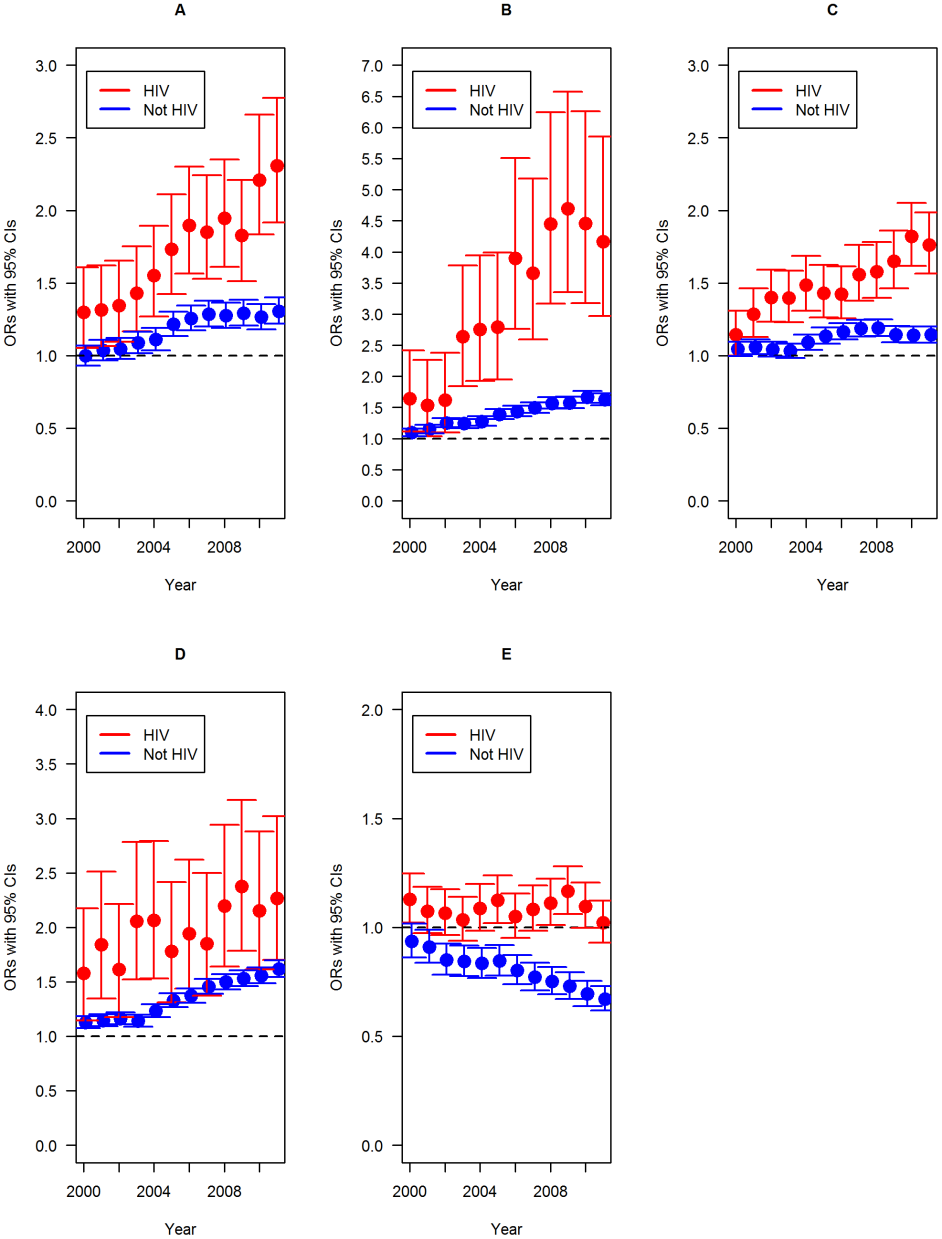
Odds ratios and 95% confidence intervals comparing the chance of having the disease listed on the death certificate over time of Non-HIV related cancers (A), external causes (B), cardiovascular diseases (C), Diabetes Mellitus (D) and Tuberculosis (E); year as a categorical variable and 1999 as the baseline reference.

The adjusted average increases for non-HIV related cancers were 6.0% (95%CI = 1.05–1.07; p<0.001) and 3.0% (95%CI = 1.02–1.03; p<0.001) per year in the HIV and non-HIV groups, respectively. Compared to 1999, the ORs for having non-HIV related cancers listed on the DC in 2011 were 2.31 (95%CI = 1.92–2.77; p<0.001) for the HIV group and 1.31 (95%CI = 1.22–1.40; p<0.001) for the non-HIV group (Figure 1A).

For external causes, there were adjusted average increases of 12.0% (95%CI = 1.09–1.15; p<0.001) and 4.0% (95%CI = 1.04–1.05; p<0.001) per year in the HIV and non-HIV groups, respectively. In comparison to 1999, the ORs for having external causes mentioned on the DC in 2011 were 4.17 (95%CI = 2.97–5.85; p<0.001) and 1.63 (95%CI = 1.53–1.72; p<0.01) for the HIV and non-HIV groups, respectively (Figure 1B).

The adjusted average increases for CVD were 4.0% (95%CI = −1.03–1.05; p<0.001) and 1.0% (95%CI = −1.00–1.02; p<0.001) per year in the HIV and non-HIV groups, respectively. Compared to 1999, the ORs for having CVD listed on the DC in 2011 were 1.76 (95%CI = 1.56–1.98; p<0.001) and 1.14 (95%CI = 1.09–1.20; p<0.001) for the HIV and non-HIV groups, respectively (Figure 1C).

DM had adjusted annual increases of 4.1% (95%CI = 1.02–1.06; p<0.001) and 3.9% (95%CI = 1.03–1.04; p<0.001) per year in the HIV and non-HIV groups, respectively. Compared to 1999, the ORs for having DM listed on the DC in 2011 were 2.27 (95%CI = 1.70–3.02; p<0.001) for the HIV group and 1.62 (95%CI = 1.54–1.70; p<0.001) for the non-HIV group (Figure 1D).

In contrast, for TB there was an adjusted average increase of 0.3% (95%CI = 1.00–1.01; p = 0.42) per year in the HIV group and a 3.0% decrease (95%CI = 0.97–0.978; p<0.001) for the non-HIV group. Compared to 1999, the ORs for having TB listed on the DC in 2011 were 1.02 (95%CI = 0.93–1.12; p = 0.66) and 0.67 (95%CI = 0.62–0.73; p<0.001) per year for the HIV and non-HIV groups, respectively (Figure 1E).

Interactions between year as a continuous or a categorical variable and HIV were both significant (p<0.001) for all conditions except for DM when year was considered as a continuous variable (p = 0.76).

## Discussion

We were among the first to describe the emergence of non-HIV related conditions as important causes of death in HIV-infected individuals in a developing country setting [1], [4], [9]. We now describe a continuous and significant increase in the proportion of non-AIDS related conditions as causes of death in HIV-infected persons from 1999 to 2011. DM, CVD, non-HIV related cancers, and external causes increased significantly more in the HIV group than in the non-HIV group. In contrast, there was a sustained decrease of TB as a cause of death in the non-HIV population, while remaining virtually unchanged in the HIV group. The latter is in keeping with reports from developed and developing country settings, where TB remains an important cause of morbidity and mortality in HIV-infected individuals [6], [10], [11].

Changes in patterns of causes of death in HIV infected individuals are mostly ascribed to the widespread availability of ART [12]–[14]. Although effective use of ART is associated with decreased incidence of conditions associated with advanced immune deficiency, a significant number of successfully treated individuals remain in a pro-inflammatory state, which in turn has been associated with non-AIDS related conditions, particularly CVD [15], [16].

There are data documenting a global increase of CVD in the HIV-infected population [1], [9], [17]. It is assumed that the prevalence of CVD is higher among age-matched HIV-infected individuals as a result of a complex interplay between a higher frequency of well-established risk factors, HIV-related inflammatory and immunologic changes, and the adverse effects of certain antiretroviral drugs. Smoking, the most important risk factor for CVD and lung cancer, is more common in HIV-infected individuals than in the general population [17] and may explain our findings at least partially.

As life expectancy increases among people living with HIV/AIDS, a growing number of individuals are at risk of co-morbid conditions that typically occur at older ages, including cancers and DM [5], [18], [19]. As an example, we have recently reported an increase in the incidence of both non-HIV and HIV-related cancers in HIV-infected in Rio de Janeiro, with lung cancer leading the former group [4]. Given the relatively high prevalence of co-infections with hepatitis B and C viruses in Brazil [20], one can expect an increasing importance of hepatic cancers as a cause of morbidity and mortality as HIV individual live longer. The significantly faster growth of external causes as causes of death in the HIV-infected population, which may be partially attributed to life style [21], is a matter that we believe deserves further investigation.

In Brazil, as elsewhere, the metabolic syndrome is increasingly common in HIV infected individuals and may play an important role in the faster growth of DM and DCV as causes of death in the HIV infected population in comparison to the general population [22].

It should be noted that our findings cannot be attributed to faster aging of the HIV-infected population alone. Even though the mean age of death in the general population increased less than the mean age of death in the HIV group (0.24% vs. 0.94% per year), all the models used were controlled for age. Moreover, it has been estimated that in Brazil two thirds of the HIV-infected individuals who died in the post-HAART era were aged 30 to 49 years [23].

A major strength of our study is that we analyzed all DC issued nationwide for a period spanning over one decade. Another strength is the use of any mention of conditions on the DC, which overcomes one of the limitations of the current ICD system, which does not cover some diseases associated with HIV.

Our study has several limitations, since we analyzed data from DC, which may lack sensitivity and specificity for certain medical conditions. Nonetheless, by using a strategy that is commonly utilized in studies that investigate occupational hazards, we were able to estimate the odds ratios by comparing individuals who had HIV/AIDS cited in their DC to those who did not. As is the case for all population-based studies, particularly those involving only death certificates data, we cannot rule out the influence of unmeasured confounders potentially associated with non-HIV associated causes of death, including smoking habits and hepatitis B and/or C serostatus. Nonetheless, we have previously reported a significant underreporting of HIV/AIDS as a cause of death in individuals known to be HIV-infected in Rio de Janeiro [24]. Predictors of underreporting included male gender, older age, and higher CD4 counts, all of which are associated with CVD and DM.

## Conclusions

The continuous increase in the frequency of non-AIDS related causes of death described in the present study might become an even greater burden for health systems as the HIV-infected population ages. On the other hand, some of these conditions, particularly CVD, DM, and lung cancer, can be prevented or effectively managed by public health interventions, such as smoking cessation and lipids, glucose, an blood pressure control. Given the potential role played by unmeasured confounders, further studies are necessary to address and elucidate the results present herein. Finally, despite the availability of effective preventative interventions, TB remains a major cause of death among HIV-infected patients. Its prevention should be a major focus of public health interventions, including wider use of isoniazid primary prophylaxis when indicated.
